# A valid question: Could hate speech condition bias in the brain?

**DOI:** 10.1093/jlb/lsw009

**Published:** 2016-03-15

**Authors:** Gail B. Murrow, Richard Murrow

**Affiliations:** ^1^Departments of Neurology and Neurosurgery, University of North Carolina at Chapel Hill, NC 27599-7025, USA

**Keywords:** dehumanization, hate speech, neuroscience, implicit bias, empathy

Here, we respond to three peer commentaries on our paper, ‘A hypothetical neurological association between dehumanization and human rights abuses’.^1^ In that paper, we hypothesized that dehumanizing implicit biases dampen the response of neural mechanisms of pain empathy to (and thereby reduce empathy for) implicitly subordinated or dehumanized targets, perhaps offering some insight into the association of dehumanization with human rights abuses.^2^ The commentaries we respond to are by Professor Robert Mark Simpson,^3^ Judge Morris B. Hoffman,^4^ and Dr Alexandra Roginsky and Professor Alexander Tsesis.^5^ We thank them all for their time and input.

The commentators’ have five general criticisms of our paper. First, Professor Simpson and Judge Hoffman argue that it implicitly or explicitly supports policy or law restricting hate speech. Second, Judge Hoffman questions the science and existence of ‘implicit bias’ and, in particular, questions a certain form of implicit racial stereotype. Third, the judge, Roginsky, and Tsesis argue that the neuroscience we rely on does not support our hypothesis. Fourth, Roginsky and Tsesis protest that our hypothesis exonerates those who commit crimes against the dehumanized. Fifth, Simpson, Roginsky, and Tsesis validly argue that we have not demonstrated a causal empirical link between hate speech and dehumanization, while the judge claims that such neuroscientific insights into how hurtful such speech may be will never justify restricting its First Amendment protection.

Our short answer is the following. We stated that our hypothesis alone was not a sufficient basis for policy recommending legal restriction of hate speech, that the neuroscience we relied on was limited and inconclusive, and that our hypothesis was theory and not scientifically proven nor agreed upon. However, the science we have cited is good science, including the neuroscience and the psychological science of implicit bias, and such science reasonably applies to and supports our hypothesis, if not proving it. Our hypothesis neither intentionally nor inadvertently excuses those who harm dehumanized others out of lack of empathy. Finally, we agree with the commentators that the next and real question is whether hate speech could ‘cause’ the dehumanizing implicit bias that we propose reduces empathy.

Regarding the first criticism, Simpson and Judge Hoffman view our paper as suggesting that its hypothesis provides support for public policy or law restricting hate speech. However, we made clear that, ‘we do not contend [] our hypothesis, as such and alone, is a sufficient basis for specific policy recommendations regarding whether various legal restrictions on … hate speech would be effective’.^6^ This quote from our paper's conclusion is the only place we use the word, ‘policy’, though the judge maintains that we have disingenuously^7^ ‘proposed that neuroscience may answer some difficult *public policy* questions’^8^ and have thereby ‘done a disservice to the integrity of science, [and] to the ideal of neutral law’.^9^ Simpson, by contrast, suggests that our claim to offer mere theory is ‘a subtle mischaracterization of what [our] analysis can and does achieve’.^10^ Both authors are also likely reacting to the concern that, not we, but other hate speech scholars, might prematurely interpret our hypothesis as sufficient to justify restricting hate speech.

We repeat that we declined to make any such policy proposals based upon our hypothesis, as that would be premature, and submit that the judge's questioning of our academic integrity in high-flown rhetoric is neither warranted nor appropriate in serious, scientific discourse.

Proceeding to the second criticism, while our hypothesis proposes that implicit bias, which dehumanizes or subordinates target social categories or groups, may automatically reduce empathy for such groups, Judge Hoffman questions both the existence and psychological science of implicit bias, specifically the reliability of one psychological tool used to test for such bias, the Implicit Associations Test (IAT) as well as evidence that such bias is linked to discriminatory responses.^11^ However, a vast body of research suggests that implicit bias is real, widespread, and impacts both affect/prejudice and cognition/stereotypes which are further linked to discriminatory responses.^12^ Further, other research methods, besides the IAT, such as functional magnetic resonance imaging (*f*MRI), have been used to provide evidence of implicit bias.^13^

We disagree with Judge Hoffman's following criticism of our remarks on implicit racial bias: ‘The Murrows label the association of African Americans with crime a ‘stereotype’, *Id.* at 6, but the sad fact is the stereotype is true, at least when it comes to violent crime … : homicide and robbery’.^14^ We maintain that statistics, such as those the judge has cited in support of this view, are not reliable evidence of crime rates, because they represent ‘*arrest* rates and not *conviction* rates’,^15^ and because of growing, recent evidence that such statistics may be skewed by racial bias implicit in the criminal justice system.^16^

While the judge has written elsewhere that ‘this idea that teeming racism has hijacked our criminal justice system is not only nonsense, it is interfering with meaningful reform’,^17^ we argue that mounting evidence of racial bias in large city ‘police practices’ (eg Ferguson, Baltimore, New York, Chicago, and San Francisco) suggests that statistics which reflect racial disparities in ‘arrests*’*, could, conversely, be viewed as suggesting that implicit, as well as explicit, racial bias does exist, permeates police practices, and undercuts the proposed accuracy of the subject racial stereotype.

The third general criticism, that the neuroscience we cite does not support our hypothesis, is made by Judge Hoffman and Roginsky and Tsesis. First, let us show how each commentary has misread our hypothesis in certain ways. For example, the judge contends that ‘The Murrow's penultimate hypothesis [is]—that dehumanization is caused by reduced empathy’.^18^ However, our hypothesis is that ‘reduced empathy’ may be ‘caused by dehumanization’,^19^ not the other way around, as the judge has described it. He has also misread our paper as proposing that humans only feel pain empathy for conspecifics,^20^ meaning members of one's own species; when what we've proposed is that empathic, or mirror neural, responses are ‘more robust’ for conspecifics.

Next, Roginsky and Tsesis have misread our hypothesis as proposing ‘that neurology is a sufficient explanation of dehumanization’.^21^ However, what we have proposed is a neurological hypothesis for how dehumanized perception might *‘*dampen pain empathy’, not a ‘neurological’ explanation for *‘*how dehumanized perception is represented or conditioned in the brain’,^22^ which is a different question we seek to address in paper in progress.

Judge Hoffman, Roginsky, and Tsesis all argue that we rely too much on mirror neuron research and theory, but we also rely on functional imaging of pain empathy.^23^ Further, though our hypothesis contemplates that ‘empathy’, broadly defined, may be modulated by some form of ‘human mirroring system’, we emphasized that this system may, or may not, involve or be limited to mirror neurons per se and most likely includes neural components yet to be defined.^24^

We informed readers that, ‘[a]lthough it has been widely assumed that mirror neurons exist in humans, there is currently very little *direct* evidence that they do…’^25^. However, the judge, while first criticizing our ‘overreliance’ upon mirror neuron research,^26^ next, criticizes this statement of ours (that there is very little direct evidence people have such neurons) as a concomitant ‘underreliance’ on mirror neuron research, or specifically as either an improper interpretation or ignorance of a specific study which he interprets as confirming that humans have such neurons.^27^

Specifically, he claimed that a study by Mukamel et al.,^28^ ‘demonstrated in 2010 that humans have mirror neurons’.^29^ Although we did not cite this specific study, we are aware of it, and, though it does provide some interesting ‘direct’ evidence (ie data from single-neuron recordings) that mirror neurons may exist in humans, it does not change our stated view that there is still ‘currently very little direct evidence that they do’.^30^ We believe this is the view of most neuroscientists. Professor Gregory Hickok wrote, in 2014, that he believes ‘it is virtually a given that humans have mirror neurons’,^31^ but that ‘[d]irect evidence of their existence is thin’.^32^

Roginsky and Tsesis assert that our hypothesis relies too much on research investigating motor mirror neurons in monkeys,^33^ and that it is too great a cross-species extrapolation to apply the above evidence from monkeys, taken along with other research of mirroring mechanisms in humans using *f*MRI, to uphold our hypothesis.^34^ Regarding studies using single-cell recordings in monkeys, they write that ‘[s]uch studies have not been, and ethically cannot be, conducted in humans’.^35^ However, some studies in humans, using single-neuron recordings, have been done in a clinical neurosurgical context and have, as Judge Hoffman indicated, revealed some direct evidence of neurons with mirroring properties in humans.^36^ These cited studies, by necessity, did not involve recording from the exact areas theorized to be the homologs of the anatomical regions in the monkey brain in which motor mirror neurons were discovered.^37^ The studies were ethical because they were done as part of a therapeutic neurosurgical procedure and thus did not significantly increase the risk to patients.^38^ For the reader's information, single-neuron recordings are routinely performed in humans as part of therapeutic neurosurgical interventions.

Roginsky and Tsesis also suggest that mirror neurons have been studied only in relation to the mirroring of motoric action, action understanding, or motor learning.^39^ However, mirror neuron research has also been applied to explain ‘empathy’.^40^ They further argue that ‘[t]he original authors of the scientific papers on mirror neurons described them as part of a motor function process, not a theory to help explain the darkest times of history and human behavior’.^41^ However, some such original authors have since theorized that neurons with mirroring properties may underpin not only motor function and action understanding, but pain empathy.^42^ We posit that lack of such pain empathy likely had a role in such dark historic rights abuses and genocide.

Finally, while Roginsky and Tsesis, in criticizing our hypothesis, contend, with respect to a Nazi's crimes, that ‘what is important is not a *motor neuron-centered* explanation of reflexive behaviors’,^43^ we reiterate that we did not claim that the ‘mirror system’ we posit is involved in pain empathy depends on the same ‘motor’ mirror neurons found in the monkey brain (or that may theoretically exist in anatomical human homologs). Instead, we propose that the human brain has a like capacity to mirror or neurally simulate the emotional components of the ‘pain’ of others, creating a sense of ‘empathy’, rather than to simply reflexively simulate, imitate, or understand ‘motoric acts*’*.

The fourth general criticism of our paper is also posed by Roginsky and Tsesis who propose that our ‘claim that neural mechanisms sufficiently explain the blunting of empathy … inadvertently exonerates the perpetrators’.^44^ We did not intend to claim or inadvertently suggest (nor do we believe did Hannah Arendt) that, because some ‘automatic’ or nonconscious neural mechanism might impact discrimination, violence, or human rights abuses, this exonerates the perpetrators of such crimes from social or moral responsibility, prosecution, or civil liability.

Though much literature in law and neuroscience, as well as the use of neuroscience in the courtroom, involves questioning the ‘criminal responsibility’ of those who engage in antisocial behavior,^45^ our hypothesis, which is that dehumanizing implicit bias dampens pain empathy for targets in humans with normal brain function, does not purport to suggest, nor offer evidence to suggest, that this seemingly common historical phenomenon prevents a finding of competence, intent, or mens rea in people who both lack empathy for ‘and’ commit crimes against dehumanized targets.

The fifth general criticism is a valid one and is best expressed by Simpson who argues that we have not provided sufficient empirical evidence that ‘hate speech’ plays a ‘causal’ role in dehumanized perception, implicit or explicit, and thus in the danger such dehumanization poses, and that such evidence would be vital in considering general legal restrictions on hate speech.^46^

Roginsky and Tsesis pose this criticism differently. In arguing that our hypothesis does not explain dehumanization, which it was not intended to do, they do go on to forcefully argue that history suggests that dehumanization is ‘caused’ by ‘socialization’ which they attribute, in significant part, to hateful rhetoric, negative stereotypes, and vilifying historical tropes. They persuasively argue that such social linguistic practice is at the real, practical root of the problem of lack of pain empathy for and discrimination and violence against targets.

We agree that if our hypothesis is correct, and dehumanizing implicit biases reduce the pain empathy for targets that discourages violation of their human rights, then the ‘next practical question’ is, ‘How are such dehumanizing implicit biases created or reinforced in the brain?’ We agree with Roginsky and Tsesis that biased language, such as hate speech, likely plays a strong hand in this. However, we also agree with Simpson that, beyond work in psychology, sociology, and linguistics that we have cited, hard empirical data to support this theory have not been shown.

We are currently reviewing the cognitive neuroscience of language for such data, and though this neuroscience is young and controversial, we will argue in a paper we are now writing that such neuroscience does suggest that biased language could condition implicit associations between targets and subhuman traits, and that such conditioning could take place automatically in the course of the non-conscious, neural processing of such linguistic stimuli and despite the hearers’ or readers’ opposing conscious beliefs, personal experience, and sensory perception.

Judge Hoffman suggests that neuroscience can ‘never’ justify antihate speech law.^47^ We argue that constitutional scholars should never thus dismiss neuroscience. However, let us clarify our position that such science does not yet offer clear answers and that, even if it did, eventually, offer clear evidence that hate speech leads to dehumanized perception and reduced pain empathy, that would not establish that there is a practical, constitutional way to legally prevent such speech or it effects, though it would increase the moral urgency to find some form of remedial response.

## Footnotes

1 Gail B. Murrow & Richard Murrow, *A Hypothetical Neurological Association between Dehumanization and Human Rights Abuses*, J. L. & Biosci. (2015), http://jlb.oxfordjournals.org/content/2/2/336.full.pdf+html

2 Id.

3 Robert M. Simpson, Commentary, *Dehumanization: Its Operations and Its Origins*, J. L. & Biosci., http://jlb.oxfordjournals.org/content/early/2015/10/16/jlb.lsv040.full.pdf+html (accessed Oct. 16, 2015).

4 Morris B. Hoffman, Commentary, *Neuroscience Cannot Answer These Questions: A Response to G. and R. Murrow's Essay Hypothesizing a Link Between Dehumanization, Human Rights Abuses and Public Policy*, J. L. & Biosci, http://jlb.oxfordjournals.org/content/early/2015/10/29/jlb.lsv041.full.pdf+html (accessed Oct. 29, 2015).

5 Alexandra B. Roginsky & Alexander Tsesis, Commentary, *Hate Speech, Volition, and Neurology*, J. L. & Biosci., http://jlb.oxfordjournals.org/content/early/2016/01/11/jlb.lsv058.full.pdf+html (accessed Dec. 16, 2015).

6 Murrow & Murrow, *supra* note 1, at 29.

7 See Hoffman, *supra* note 4, at 4.

8 *Id.* (emphasis added).

9 *Id.* at 7.

10 Simpson, *supra* note 3, at 6.

11 See Hoffman, *supra* note 4, at 3, n.11.

12 See eg David M. Amodio & Patricia G. Devine, *Stereotyping and Evaluation in Implicit Race Bias: Evidence for Independent Constructs and Unique Effects on Behavior*, 91(4) J. Pers. and Soc. Psych. 652, 661 (2006).

13 Murrow & Murrow, *supra* note 1, at 7, n.20, n.22.

14 Hoffman, *supra* note 4, at n.14.

15 Id.

16 See eg U.S. Dep't of Justice, Investigation of the Ferguson Police Department, 4, 62–78 (Mar. 4, 2015), http://www.justice.gov/sites/default/files/opa/press-releases/attachments/2015/03/04/ferguson_police_department_report.pdf (accessed Jan. 30, 2016).

17 See Morris B. Hoffman, Opinion, *Too Many Prison Inmates: It's Not So Simple*, Denver Post, Nov. 15, 2014, http://www.denverpost.com/opinion/ci_26938368/too-many-inmates-its-not-so-simple. But see Nathan Woodliff-Stanley, Letter to the Editor, *Is There Racial Bias In U.S. Prison sentencing?* Denver Post, Nov. 23, 2014, http://blogs.denverpost.com/eletters/2014/11/22/racial-bias-u-s-prison-sentencing-2-letters/34797/

18 Hoffman, *supra* note 4, at 4.

19 See Murrow & Murrow, *supra* note 1, at 2.

20 See Hoffman, *supra* note 4, at 2.

21 Roginsky & Tsesis, *supra* note 5, at 1.

22 See Murrow & Murrow, *supra* note 1.

23 *Id.* at 11, 12, n.36.

24 *Id.* at 9, 10–2.

25 *Id.* at 10 (emphasis added).

26 Hoffman, *supra* note 4, at 2, 3.

27 Id.

28 Roy Mukamel et al., *Single-Neuron Responses in Humans during Execution and Observation of Actions*, 20 Curr. Biol. 750, 756 (2010).

29 Hoffman, *supra* note 4, at 3.

30 Murrow & Murrow, *supra* note 1, at 10.

31 Gregory Hickok, The Myth of Mirror Neurons: The Real Neuroscience of Communication and Cognition 27 (2014).

32 Id.

33 Roginsky & Tsesis, *supra* note 5, at 2, 3.

34 See *id.*

35 *Id.* at 2.

36 See Mukamel et al., *supra* note 26, at 750–756; William D. Hutchinson et al., *Pain-Related Neurons in the Human Cingulate Cortex*, 2 Nature Neurosci. 404 (1999).

37 See *id.*

38 Id.

39 See Roginsky & Tsesis, *supra* note 5, at 2, 3.

40 See Murrow & Murrow, *supra* note 1, at 9, n.27, 10, 12.

41 Roginsky & Tsesis, *supra* note 5, at 4.

42 See Giacomo Rizzolatti, Leonardo Fogassi & Vittorio Gallese, *Mirrors In The Mind: A Special Class Of Brain Cells Reflects The Outside World, Revealing A New Avenue For Human Understanding, Connecting And Learning*, Scientific American, Nov. 2006, at 62.

43 Roginsky & Tsesis, *supra* note 5, at 3 (emphasis added).

44 *Id.* at 3.

45 See eg Uri Maoz & Gideon Yaffe, *What Does Recent Neuroscience Tell Us About Criminal Responsibility?* J. L. & Biosci. (Advance Access Online Publication Dec. 30, 2015), http://jlb.oxfordjournals.org/content/early/2015/12/30/jlb.lsv051.full.pdf+html.

46 See Simpson, *supra* note 3, at 5.

47 See Hoffman, *supra* note 4, at 6.

